# Cigarette smoking and thyroid cancer risk: A Mendelian randomization study

**DOI:** 10.1002/cam4.6570

**Published:** 2023-09-25

**Authors:** Hongzhan Jiang, Yi Li, Jiali Shen, Huihui Lin, Siyue Fan, Rongliang Qiu, Jiaxi He, Ende Lin, Lijuan Chen

**Affiliations:** ^1^ School of Nursing Fujian University of Traditional Chinese Medicine Fuzhou China; ^2^ The School of Clinical Medicine Fujian Medical University Fuzhou China; ^3^ School of Medicine Xiamen University Xiamen China; ^4^ Department of General Surgery Zhongshan Hospital of Xiamen University, School of Medicine Xiamen China

**Keywords:** causality, Mendelian randomization, smoking, thyroid cancer

## Abstract

**Background:**

The association between cigarette smoking and thyroid cancer has been reported in prospective cohort studies, but the relationship remains controversial. To investigate this potential correlation further, we employed Mendelian randomization methodology to evaluate the causative impact of smoking on thyroid cancer incidence.

**Methods:**

From the genome‐wide association study and Sequencing Consortium of Alcohol and Nicotine use, we obtained genetic variants associated with smoking initiation and cigarettes per day (1.2 million individuals). We also extracted genetic variants associated with past tobacco smoking from the UK Biobank (424,960 individuals). Thyroid cancer outcomes were selected from the FinnGen GWAS (989 thyroid cancer cases and 217,803 control cases). Sensitivity analyses employing various approaches such as weighted median, MR‐Egger, and MR‐pleiotropy residual sum and outlier (MR‐PRESSO) have been executed, as well as leave‐one‐out analysis to identify pleiotropy.

**Results:**

Using the IVW approach, we did not find evidence that any of the three smoking phenotypes were related to thyroid cancer (smoking initiation: odds ratio (OR) = 1.56, *p* = 0.61; cigarettes per day: OR = 0.85, *p* = 0.51; past tobacco smoking: OR = 0.80, *p* = 0.78). The heterogeneity (*p* > 0.05) and pleiotropy (*p* > 0.05) testing provided confirmatory evidence for the validity of our MR estimates.

**Conclusions:**

The MR analysis revealed that there may not exist a causative link between smoking exposure and elevated incidence rates of thyroid malignancies.

## Abstract 2

We used Mendelian randomization analysis to evaluate the causal relationship between smoking and thyroid cancer, and found that smoking was not associated with thyroid cancer.

## 1 INTRODUCTION

In most parts of the world, thyroid cancer incidence has significantly grown during the last few decades.^1^ In the United States, thyroid carcinoma ranks 13th in terms of newly diagnosed tumors in the United States each year, with over 440,000 forecasted instances in 2022.^2^ According to their pathogenesis, histopathological features, and clinical presentation, thyroid cancer are categorized into four main subtypes: differentiated (follicular or papillary) thyroid 999 cancers, poorly differentiated thyroid cancers, anaplastic thyroid cancers, and medullary thyroid cancers.^3^ Differentiated cancers, which have a usually good prognosis, account for 80% of all thyroid cancers.^4^ Thyroid cancer has been recognized as a cancer type with a significant related financial burden on patients.^5^ The risk of thyroid cancer seems to be increased by some factors such as race, radiation exposure, and women, but no controllable risk factors have been identified for thyroid cancer.^6^ To further reduce thyroid cancer burden and prevalence, we should pay more attention to other controllable risk factors, such as cigarette smoking.^7^

Smoking is one of the leading causes of various types of cancer, including colorectal, prostate, lung, stomach, and cervix cancers.^8^ However, the correlation between smoking and thyroid carcinoma remains elusive. Some studies show that smoking has a carcinogenic effect on thyroid cancer.^9^ The negative relationship between smoking and thyroid cancer was reported in a cohort study that followed 96,855 adults for 5.9 years.^7^ A meta‐analysis indicated that smokers have a lower risk of thyroid carcinoma (OR = 0.798; 95% CI = 0.681–0.935).^10^ A Korean cohort study based on population data revealed that smoking exerts a protective effect against thyroid cancer.^11^ Other studies showed that smoking does not significantly affect thyroid cancer.^12^ Whether smoking is a causal factor in the emergence of thyroid cancer is still up for disagreement due to the contradictory findings from several independent studies.

To clarify the direction and strength of the association between smoking behavior and thyroid cancer, we implemented the MR analysis, a powerful tool from genetic epidemiology that uses genetic polymorphisms as instrumental variables (IVs) to minimize sources of bias stemming from confounding or reverse causation.^13^ By applying this approach to disentangle the causal linkages between smoking exposure and thyroid malignant tumors, we aim to shed new light on the prevention of thyroid cancer.

Our investigation utilized an MR approach to evaluate the putatively causal connection between cigarette smoking and the likelihood of developing thyroid carcinomas by leveraging published large‐scale genome‐wide association studies (GWAS) data sets relating to varying smoking characteristics (smoking initiation, cigarettes per day, and past tobacco use).

## 2 METHODS

### 2.1 Genetic variants associated with smoking

**FIGURE 1:**
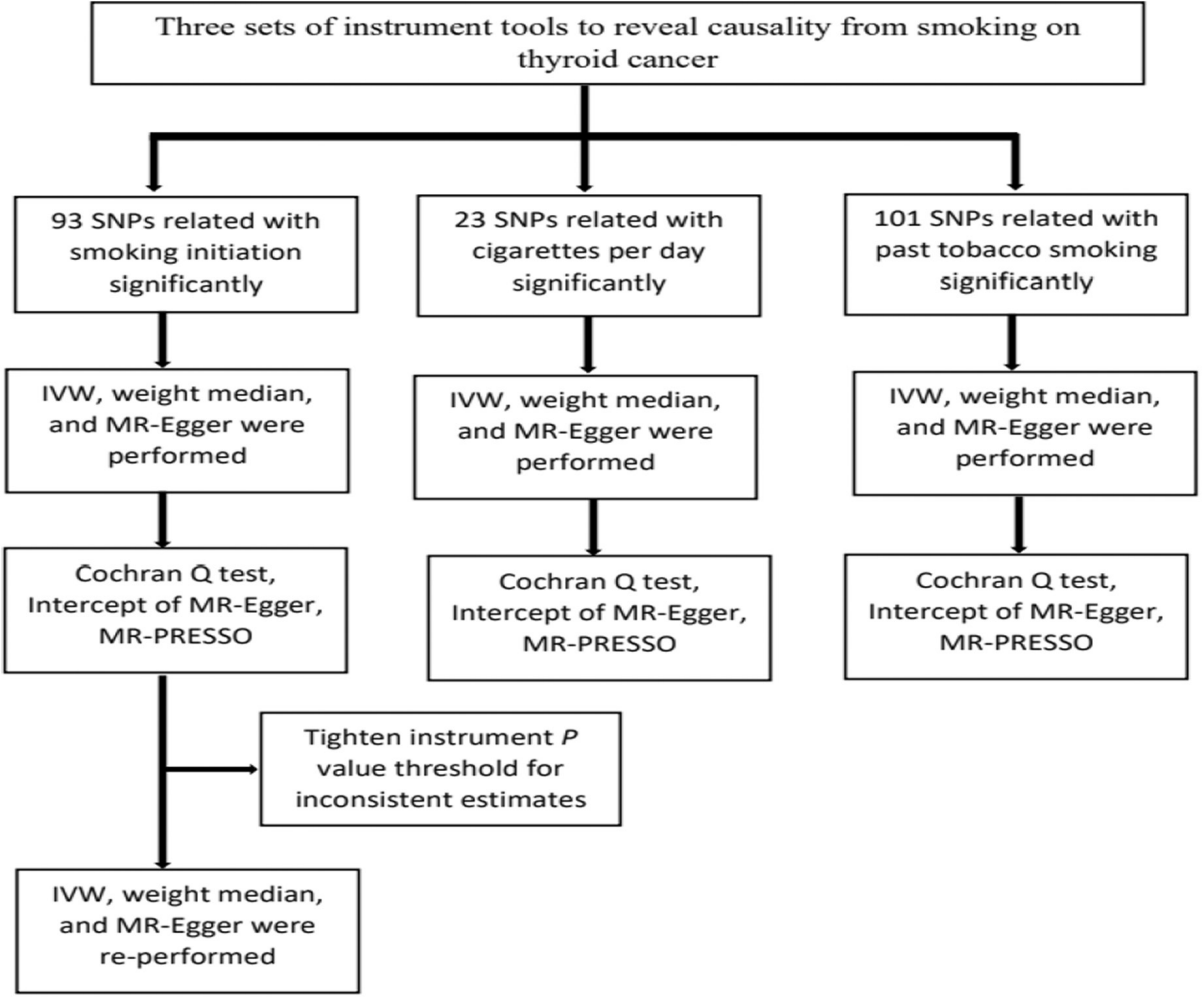
Workflow of the MR study demonstrating the link between thyroid cancer and smoking. IVW, inverse variance weighted; MR, Mendelian randomization; MR‐PRESSO, MR Pleiotropy Residual Sum and Outlier; SNP, single‐nucleotide polymorphisms.

The GWAS summary statistics of tobacco use were extracted from the UK Biobank and the GWAS and Sequencing Consortium of Alcohol and Nicotine use (GSCAN) for three smoking phenotypes. Genetic IVs for the exposure were selected at the genome‐wide significance level (*p* < 5 × 10^−8^, linkage disequilibrium [LD] *r* ^2^ < 0.01) across a 1 Mb region.^14^ The study design of MR analysis is presented in Figure 1.

We extracted IVs of smoking initiation and cigarettes per day from the GSCAN. For smoking initiation GWAS, they included 607,291 European individuals. Smoking initiation was defined as having ever been a regular smoker in life (current or former). A total of 93 SNPs related to smoking initiation were identified (Supplementary Material, Table S1). For cigarettes per day GWAS, they included 337,334 European individuals and identified 23 significant SNPs (Supplementary Material, Table S2). For past tobacco smoking GWAS, they included 424,960 Europeans and a total of 101 SNPs were significant (Supplementary Material, Table S3). In the Supplementary Material, more information on these smoking phenotypes is available.

To assess the presence of weak instrument bias, we computed estimates of the strength of the relationship between the genetic instruments and smoking phenotypes utilizing the *F* statistic (*F* = beta^2^/se^2^). By doing so, we aimed to determine whether the instruments possess sufficient explanatory power to influence the outcome variable. The *F* statistical range of the instrument SNPs used in MR analysis is 29.8–961, exceeding the suggested *F* > 10 threshold.^15^

### 2.2 GWAS summary data for thyroid cancer

The GWAS summary data for thyroid cancer were extracted from FinnGen (https://r5.finngen.fi/), including 989 patients and 217,803 controls of European ancestry. The Cancer Registry data were used to identify cases of thyroid cancer, according to the code C73 of the International Classification of Diseases‐10.

### 2.3 MR analyses

For each instrumental variable, the Wald ratio was used to evaluate the influence of exposure on the result. Then, we used the inverse variance weighted (IVW) method to combine each instrumental variable's effect size. This method is based on the premise that instruments can only affect a result through exposure and not through any other channel.^16^ The weighted median and MR‐Egger methods were employed to supplement IVW estimates. A stricter instrument *p* value threshold was set if the estimations from the methods used in our investigation were inconsistent.^17^ All MR analysis approaches have consistent MR results, which could provide more reliable predictions in a wider range of scenarios.^18^ The MR analysis was combined using a random‐effect model.

### 2.4 Sensitivity analyses

The heterogeneity was evaluated using the Cochrane's Q value from the IVW approach, with a *p* value <0.05 indicating statistical significance.^19^, ^20^ The MR‐Egger intercept and MR‐PRESSO methods were used to detect horizontal pleiotropy (directional pleiotropy was assumed to exist if *p* < 0.05),^21^ MR‐PRESSO includes three components: (i) identification of horizontal pleiotropy; (ii) horizontal pleiotropy correction by the elimination of outliers; (iii) analyzing the causal estimates before and after the outlier correction to determine whether there are any discrepancies.^22^ A leave‐one‐out approach was employed to evaluate the effect of a particular SNP on the outcome of the MR analysis.

The genetic relationships between smoking and the risk of thyroid cancer were displayed using scatter plots and funnel plots to visually evaluate the consistency of MR estimations and any potential bias.^23^

MR analyses were performed by the R package “TwoSampleMR” (version 0.5.6) and “MRPRESSO” (version 1.0) in R (version 4.2.0).

## 3 RESULTS

### 3.1 MR Analysis of smoking with risk of thyroid cancer

**FIGURE 2:**
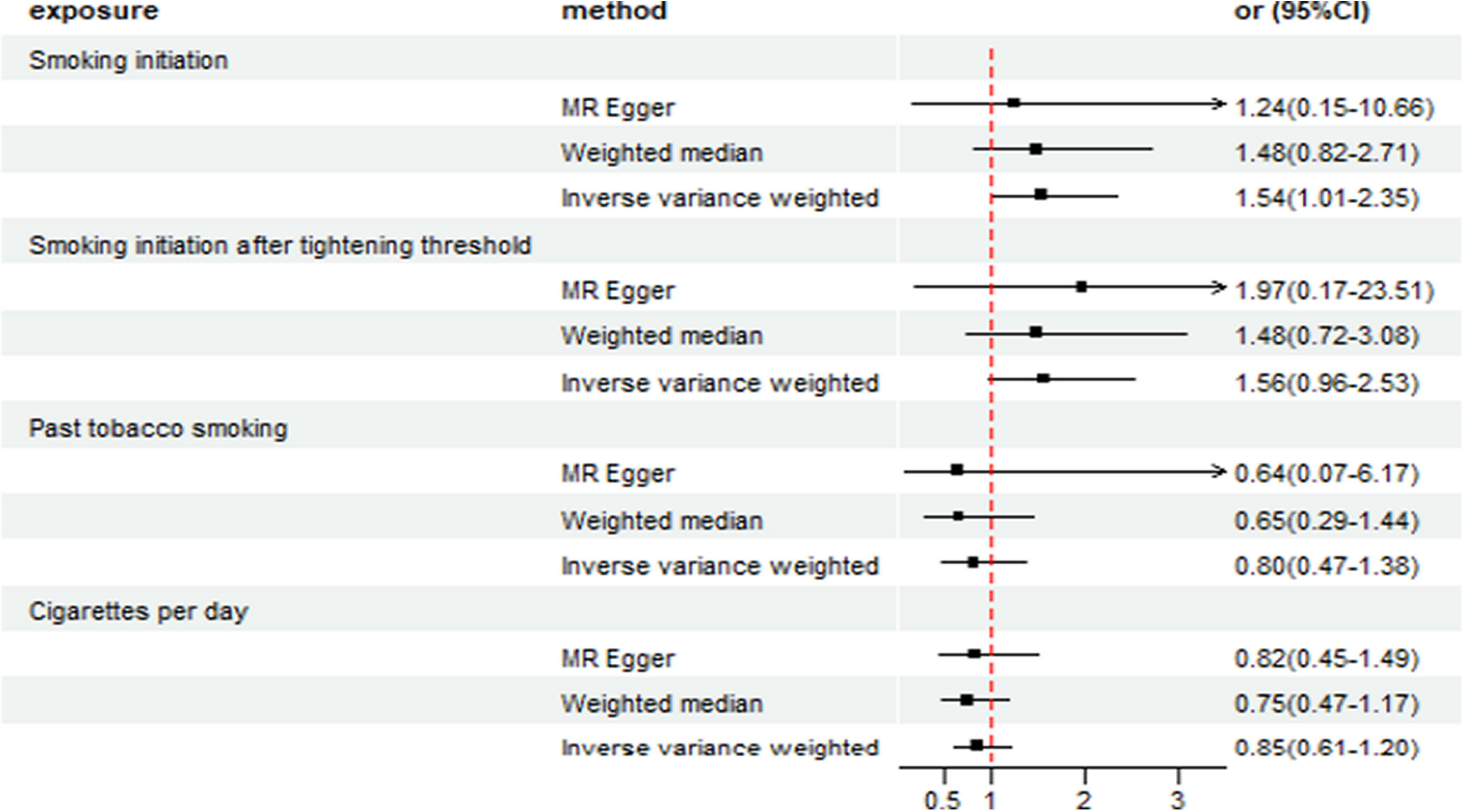
Odds ratio plot for smoking initiation, cigarettes per day, and past tobacco smoking. CI: confidence interval; OR: odds ratio.

To investigate the causal association between smoking‐related SNPs and thyroid cancer, we conducted a two‐sample MR study using genetic data from participants of European descent. Our analysis involved utilizing three different methods for estimating instrumental variables (IVs), including the IVW, WM, and MR‐Egger. By evaluating interactions between these inputs and outcomes, our goal was to assess the validity of inferring a causal relationship. In the standard IVW method, we found smoking initiation increased the risk for thyroid cancer significantly (OR = 1.54, 95% CI = 1.01–2.35, *p* = 0.047), while opposing results were observed using the MR‐Egger (OR = 1.24, 95% CI = 0.15–10.66, *p* = 0.842) and WM approaches (OR = 1.48, 95% CI = 0.82–2.71, *p* = 0.195). Since the MR estimates for IVW and MR‐Egger were incompatible, we implemented stringent criteria by setting the instrument *p* value threshold at 1 × 10^−8^. This adjustment resulted in the inclusion of 59 SNPs as instrumental variables.^14^ The MR estimations became nonsignificant, the IVW (OR = 1.56, 95% CI: 0.96–2.53, *p* = 0.070) did not detect evidence of the impact of smoking on thyroid cancer, while the MR‐Egger (OR = 1.97, 95% CI: 0.17–23.51, *p* = 0.592) also provided inconclusive findings regarding direct effects of smoking on disease risk. Finally, the WM estimate did not identify a statistically significant relationship either (OR = 1.48, 95% CI: 0.72–3.08, *p* = 0.282). No evident heterogeneity was found, as indicated by the Cochran Q‐test derived *p* value (IVW: Q = 54.29, *df* = 58, *p* = 0.61; ME‐Egger: Q = 54.26, *df* = 57, *p* = 0.58) (Table 1). We found that cigarettes per day (OR = 0.85, 95% CI = 0.61–1.20, *p* = 0.369); past tobacco smoking (OR = 0.80 95% CI = 0.47–1.38, *p* = 0.429) were not causally associated with thyroid cancer risk using IVW method. Furthermore, the results of MR‐Egger regression and WM approaches also support this finding. The MR estimates of smoking in thyroid cancer using conventional MR analysis (MR‐Egger, WM, and IVW) are presented in Figure 2.

**TABLE 1:** Mendelian randomization estimates for the association between thyroid cancer and smoking.

Smoking phenotypes	IVW			Weighted median	ME‐Egger					MR‐PRESSO
	OR (95% CI)	Cochran Q Statistics (*df*)	*p*	OR (95% CI)	OR (95% CI)	Cochran Q Statistics (*df*)	*p*	Intercept (se)	*p*	*p*
Smoking initiation	1.54 (1.01–2.35)	69.99 (84)	0.86	1.48 (0.82–2.71)	1.24 (0.15–10.66)	69.95 (83)	0.85	0.0056 (0.028)	0.84	0.86
Smoking initiation after tightening threshold	1.56 (0.96–2.53)	54.29 (58)	0.61	1.48 (0.72–3.08)	1.97 (0.17–23.51)	54.26 (57)	0.58	−0.0065 (0.034)	0.85	0.62
Cigarettes per day	0.85 (0.61–1.20)	20.09 (21)	0.51	0.75 (0.47–1.17)	0.82 (0.45–1.49)	20.07 (20)	0.45	0.0035 (0.02)	0.86	0.56
Past tobacco smoking	0.80 (0.47–1.38)	82.04 (93)	0.78	0.65 (0.29–1.44)	0.64 (0.07–6.17)	81.99 (92)	0.76	0.0046 (0.02)	0.83	0.79

MR‐PRESSO identified no outlier variants in all analyses (for smoking initiation, *p* = 0.86; for smoking initiation after tightening threshold, *p* = 0.62; for cigarettes per day, *p* = 0.56; for past tobacco smoking, *p* = 0.79) (Table 1). The MR‐Egger method's intercepts showed that there is no evidence for the potential pleiotropy of a single SNP (for smoking initiation, intercept = 0.0056, *p* = 0.84; for smoking initiation after tightening threshold, intercept = −0.0065, *p* = 0.85; for cigarettes per day, intercept = 0.0035, *p* = 0.86; for past tobacco smoking, intercept = 0.0046, *p* = 0.83) (Table 1) (Supplementary Material, Figures S1, S4, and S7). Simultaneously checked the stability of our observations using the “leave one out method” (Supplementary Material, Figures S2, S5, and S8). Additionally, the funnel plot was symmetry, showing no pleiotropy (Supplementary Material, Figures S3, S6, and S9). These evaluations confirm the reliability of our research results (Table 1).

## 4 DISCUSSION

To comprehensively assess whether smoking has a causal impact on the incidence of thyroid cancer, we used a two‐sample MR approach, and we found no conclusive evidence to support the causative role of genetically predicted cigarette smoking on the risk of thyroid cancer.

Up to now, the relationship between smoking and thyroid cancer has remained unclarified.^7^, ^10^, ^24^, ^25^ The majority of prior epidemiological research used case–control methodologies, which failed to clarify the causal relationship between thyroid cancer and smoking. Evidence on the relationship between smoking and thyroid cancer is inconsistent. A large cohort study reported that smoking was significantly associated with a lower risk of thyroid cancer,^7^ a finding confirmed by another meta‐analysis.^20^ At the same time, a population‐based Korean cohort study also indicated that smoking reduces the risk of thyroid cancer, both in men and women.^11^ However, not all research supports this conclusion. In prospective cohort studies carried out in North American populations, researchers found no statistically significant association between cigarette smoking and the incidence of thyroid cancer over a median follow‐up period spanning approximately 15–20 years. This null finding may be indicative of the limited or non‐existent influence exerted by smoking on thyroid carcinogenesis.^12^, ^26^ In light of the substantial gender disparity in thyroid cancer incidence, whereby men experience a much lower burden compared to females.^27^ Specifically, in most studies, statistical significance has been observed between current smoking habits and thyroid tumors exclusively among women rather than men.^28^, ^29^ In addition, different types of thyroid cancer vary greatly. However, previous observational studies have not examined the effects of smoking on different subtypes of thyroid cancer. Therefore, further investigation is needed to test the relationship between smoking and different subtypes of thyroid cancer.

The variability of experimental design and analysis methods adopted by different researchers may be the main reason for the differences in research conclusions on the relationship between tobacco use and thyroid malignant tumors. Additional potential sources of inconsistency encompass differences in study participant characteristics such as age, demographics, and other comorbidities; and distinctions in classification systems used to define cases of thyroid cancer. Moreover, the differences in environmental exposure, lifestyle, diagnostic techniques, medical practice, and monitoring strategies in different research environments must be considered. In summary, while past empirical works have attempted to account for possible confounding factors. However, certain unmeasured variables remain possible sources of bias.^7^, ^30^, ^31^, ^32^ Our study addressed these concerns by employing MR analysis of multiple instrumental variables, subsequently demonstrating no evidence of a causative association between cigarette smoking and incident occurrences of thyroid carcinoma. Collectively, these null findings suggest that factors other than smoking contribute substantially to thyroid cancer pathogenesis. Thyroid cancer cells could evade immune surveillance by establishing an immunosuppressive microenvironment.^33^ Furthermore, BRAF (rapidly accelerating fibrosarcoma homolog B) and RAS (rat sarcoma) gene mutations associated with impaired immunity activate the PI3K signaling pathway, enhancing cell growth, survival, and angiogenesis in thyroid cancer cells.^34^, ^35^ Future investigations are warranted to better understand the etiologic determinants of this malignancy.

Though our research indicated that there was no causality between smoking and thyroid cancer, it was possible that smoking might have a potential impact on the development of thyroid cancer. First, cigarette smoke contains thiocyanate, which impacts the thyroid by competitively inhibiting the uptake and organification of iodine in the gland.^36^ Previous research findings demonstrate that, among individuals who smoke, there exists a trend toward decreased circulatory concentrations of thyroid stimulating hormone (TSH), as well as the bioactive metabolites of thyroxine (T4) and triiodothyronine (T3).^37^, ^38^ Furthermore, it has previously been postulated that altered regulation of TSH may be pivotal in promoting thyroid cancer.^39^ Smoking may also affect the risk of thyroid cancer by changing the sex steroid hormone levels.^40^, ^41^ Thyroid cancer was also associated with autoimmune thyroiditis (mainly Graves' disease and Hashimoto's disease). Previous investigations have suggested that nicotine present in tobacco can skew dysfunctional Th1 and Th17 immune responses toward a protective Th2 response, thereby potentially reducing the likelihood of autoimmune thyroiditis.^36^ Additionally, the alkaloid compound anatabine derived from tobacco plants has been demonstrated to attenuate both the prevalence and severity of autoimmune thyroiditis.^42^

This study possesses several noteworthy advantages due to its use of extensive datasets and meticulous methodology. We leveraged the potential of MR to reduce issues related to reverse causality and confounding. By adopting this strategy, we were able to minimize these concerns compared to traditional observational approaches. Furthermore, our MR design yielded an F statistics value greater than 10, suggesting a minimal possibility of weak instrument variable bias. Additional support for the validity of our findings comes from the absence of horizontal pleiotropy among instrumental variables observed via the application of MR‐Egger intercepts and MR‐PRESSO estimations. Finally, the successful implementation of the “leave one out” technique underscores the dependable nature of our conclusions. These outcomes highlight the rigor of our methodological approach and substantiate our research findings.

However, several limitations were also present in our study. First, we limited the research to people of European ancestry, which decreased the possibility of population structure‐related bias but constrained the applicability of our findings to other populations. Second, despite the large sample size, the proportion of cases in the results is relatively low and may have an impact on MR estimation results. Finally, performing subgroup analysis is difficult when employing GWAS summary‐level data. A further study including thyroid cancer subgroups can be considered in the future.

## 5 CONCLUSION

This is the inaugural MR study to examine the correlation between smoking and thyroid cancer. Our comprehensive outcomes do not corroborate the existence of a direct association between smoking and thyroid cancer occurrence. In the future, a large GWAS dataset is necessary to scrutinize the causal connection between cigarette smoking and thyroid cancer.

## AUTHOR CONTRIBUTIONS

**Hongzhan Jiang:** Conceptualization (equal); data curation (equal); formal analysis (equal); investigation (equal); methodology (equal); resources (equal); software (equal); visualization (equal); writing – original draft (equal). **Yi Li:** Conceptualization (equal); data curation (equal); formal analysis (equal); investigation (equal); methodology (equal); resources (equal); software (equal); visualization (equal); writing – review and editing (equal). **Jiali Shen:** Conceptualization (equal); visualization (equal); writing – review and editing (equal). **Huihui Lin:** Conceptualization (equal); writing – review and editing (equal). **Siyue Fan:** Writing – review and editing (equal). **Rongliang Qiu:** Writing – review and editing (equal). **Jiaxi He:** Writing – review and editing (equal). **Ende Lin:** Writing – review and editing (equal). **Lijuan Chen:** Conceptualization (equal); funding acquisition (equal); writing – review and editing (equal).

## FUNDING INFORMATION

This research was supported by the Xiamen Municipal Science & Technology Project (No. 3502Z20209025).

## CONFLICT OF INTEREST STATEMENT

The authors declare no conflict of interest.

## ETHICS STATEMENT

We used publicly available summary‐level data. No additional patient consent and ethical approval are required.

## Supporting information


Data S1.

## Data Availability

All the data used in the current research are publicly available GWAS summary data.
