# Supplementary material for: Cigarette smoking and thyroid cancer risk: A Mendelian randomization study
Source: Cancer Med. 2023 Sep 25;12(19):19866–73. doi: 10.1002/cam4.6570 (PMC10587937; doi:10.1002/cam4.6570)
Supplement: Supplementary file 1 — Data S1. [file CAM4-12-19866-s001.docx]

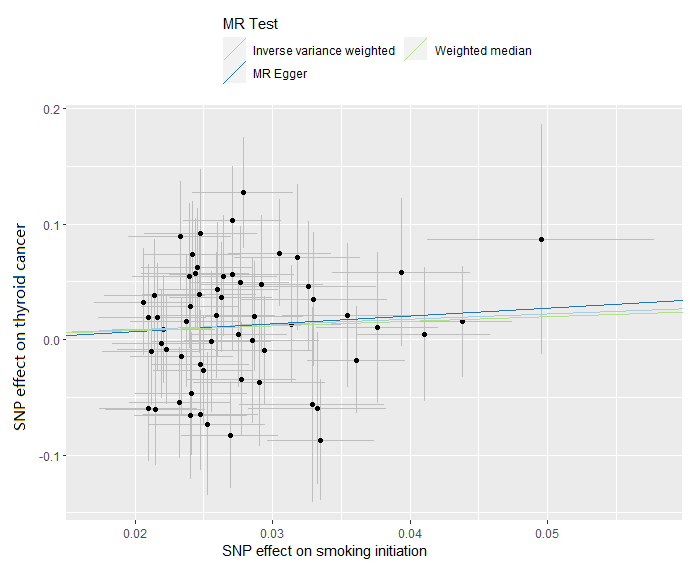


Figure S1. Scatter plot of SNPs associated with smoking initiation and their risk of thyroid cancer after tightening instrument P value threshold.


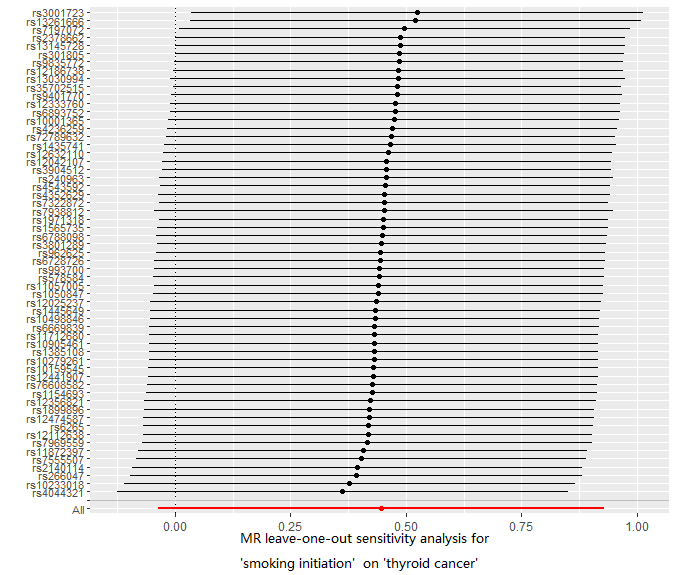
Figure S2. Leave-one-out of SNPs associated with smoking initiation and their risk of thyroid cancer after tightening instrument P value threshold.


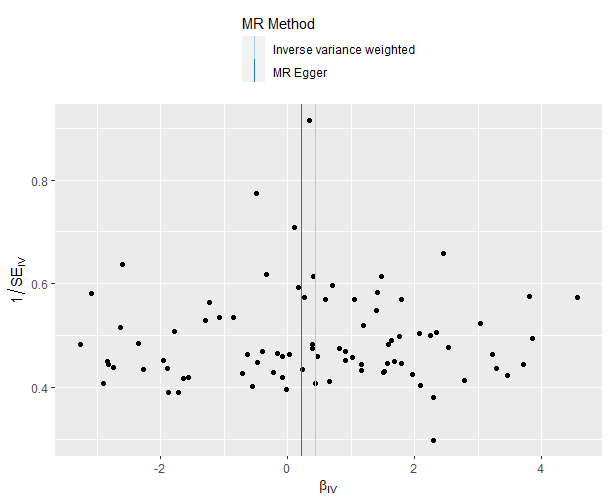


Figure S3. Funnel plot of SNPs associated with smoking initiation and their risk of thyroid cancer after tightening instrument P value threshold.


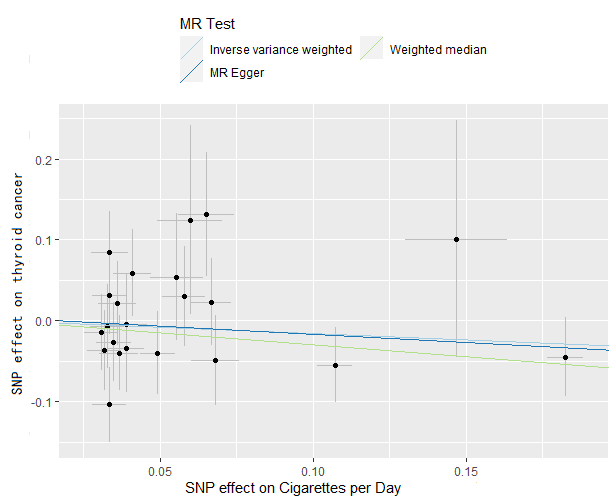


Figure S4. Scatter plot of SNPs associated with cigarettes per day and their risk of thyroid cancer.


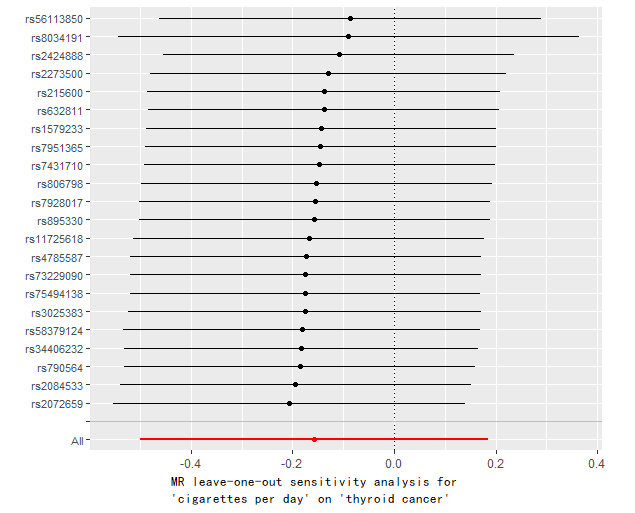


Figure S5. Leave-one-out of SNPs associated with cigarettes per day and their risk of thyroid cancer.


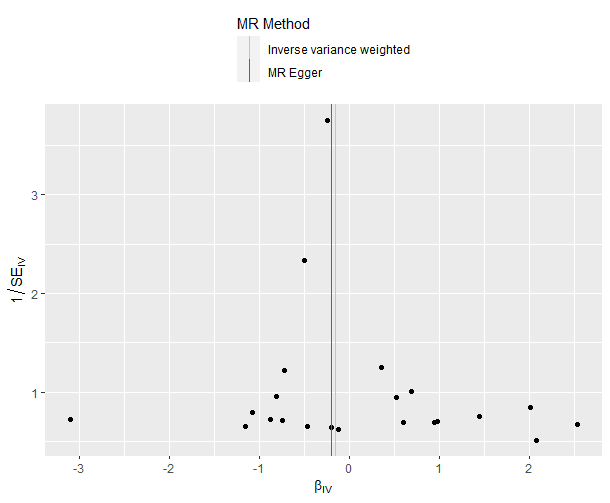


Figure S6. Funnel plot of SNPs associated with cigarettes per day and their risk of thyroid cancer.


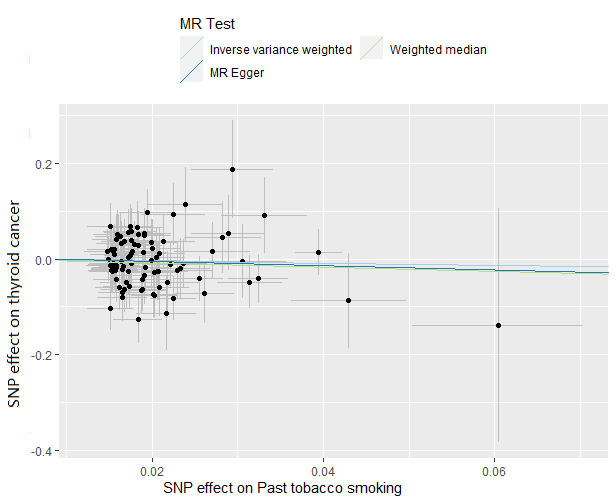


Figure S7. Scatter plot of SNPs associated with past tobacco smoking and their risk of thyroid cancer.


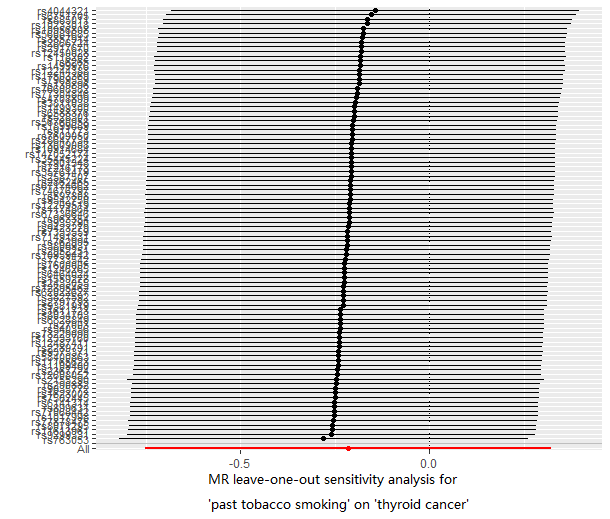


Figure S8. Leave-one-out of SNPs associated with past tobacco smoking and their risk of thyroid cancer.


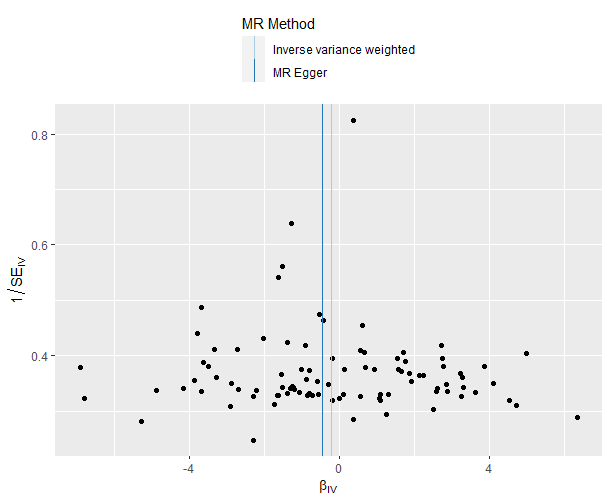


Figure S9. Funnel plot of SNPs associated with past tobacco smoking and their risk of thyroid cancer.

Supplementary Table 1. 93 SNPs associated with smoking initiation.

| SNP | Chromosome | Position | Effect  allele | Other allele | Frequency | Beta | Se | P | Sample size | F |
| --- | --- | --- | --- | --- | --- | --- | --- | --- | --- | --- |
| rs3001723 | 1 | 44037685 | A | G | 0.321 | 0.0335 | 0.0039 | 8.12E-18 | 632802 | 73.9 |
| rs7555507 | 1 | 73766037 | T | C | 0.496 | -0.0241 | 0.0036 | 1.14E-11 | 632802 | 46.1 |
| rs6669839 | 1 | 50625979 | T | C | 0.204 | 0.0260 | 0.0044 | 3.36E-09 | 632802 | 35.0 |
| rs12042107 | 1 | 91196176 | C | T | 0.527 | -0.0223 | 0.0036 | 4.22E-10 | 632802 | 39.0 |
| rs2186122 | 1 | 66470206 | T | A | 0.561 | 0.0261 | 0.0036 | 3.61E-13 | 632802 | 52.8 |
| rs301805 | 1 | 8481016 | G | T | 0.559 | 0.0215 | 0.0036 | 2.80E-09 | 632802 | 35.3 |
| rs12025237 | 1 | 154205120 | C | A | 0.124 | -0.0330 | 0.0053 | 6.52E-10 | 632802 | 38.2 |
| rs2050586 | 1 | 87905828 | C | G | 0.355 | -0.0205 | 0.0037 | 3.00E-08 | 632802 | 30.7 |
| rs2046850 | 1 | 210304319 | T | C | 0.187 | -0.0248 | 0.0045 | 3.03E-08 | 632802 | 30.7 |
| rs6728726 | 2 | 623976 | C | T | 0.829 | 0.0354 | 0.0047 | 6.73E-14 | 632802 | 56.1 |
| rs78411160 | 2 | 58171220 | C | A | 0.631 | 0.0205 | 0.0037 | 2.03E-08 | 632802 | 31.5 |
| rs6433897 | 2 | 182034448 | C | T | 0.754 | 0.0224 | 0.0041 | 3.16E-08 | 632802 | 30.6 |
| rs266047 | 2 | 104088751 | A | G | 0.529 | -0.0305 | 0.0037 | 3.36E-16 | 632802 | 66.6 |
| rs4674993 | 2 | 226332033 | G | A | 0.207 | -0.0252 | 0.0044 | 1.32E-08 | 632802 | 32.3 |
| rs578584 | 2 | 45143175 | T | A | 0.605 | 0.0287 | 0.0036 | 1.50E-15 | 632802 | 63.6 |
| rs35702515 | 2 | 137542847 | T | G | 0.162 | 0.0252 | 0.0042 | 2.43E-09 | 632802 | 35.6 |
| rs13030994 | 2 | 146143090 | A | G | 0.485 | 0.0361 | 0.0036 | 3.56E-24 | 632802 | 103.0 |
| rs12474587 | 2 | 162802993 | T | G | 0.404 | 0.0276 | 0.0036 | 1.25E-14 | 632802 | 59.5 |
| rs2107300 | 2 | 200937901 | G | C | 0.845 | -0.0272 | 0.0049 | 3.27E-08 | 632802 | 30.5 |
| rs7585579 | 2 | 60024857 | G | C | 0.505 | 0.0224 | 0.0037 | 1.88E-09 | 632802 | 36.1 |
| rs1445649 | 2 | 155682556 | C | T | 0.525 | 0.0240 | 0.0036 | 1.68E-11 | 632802 | 45.3 |
| rs6788098 | 3 | 85624131 | T | A | 0.623 | -0.0313 | 0.0037 | 1.91E-17 | 632802 | 72.2 |
| rs12632110 | 3 | 50224225 | G | A | 0.647 | -0.0234 | 0.0038 | 4.78E-10 | 632802 | 38.8 |
| rs11712680 | 3 | 75009019 | C | A | 0.174 | -0.0270 | 0.0046 | 3.51E-09 | 632802 | 34.9 |
| rs1154693 | 3 | 117804154 | G | A | 0.856 | 0.0326 | 0.0049 | 3.12E-11 | 632802 | 44.1 |
| rs66680800 | 3 | 85985324 | T | G | 0.397 | -0.0203 | 0.0037 | 2.83E-08 | 632802 | 30.8 |
| rs1869243 | 3 | 5724536 | C | T | 0.481 | 0.0197 | 0.0036 | 2.97E-08 | 632802 | 30.7 |
| rs9835772 | 3 | 85766025 | T | A | 0.235 | 0.0240 | 0.0041 | 6.32E-09 | 632802 | 33.7 |
| rs962625 | 4 | 28473524 | G | A | 0.24 | 0.0237 | 0.0040 | 4.37E-09 | 632802 | 34.5 |
| rs993700 | 4 | 67825894 | C | T | 0.766 | -0.0259 | 0.0043 | 1.53E-09 | 632802 | 36.5 |
| rs13145728 | 4 | 140927812 | C | G | 0.358 | -0.0233 | 0.0037 | 2.14E-10 | 632802 | 40.3 |
| rs10001365 | 4 | 147797214 | A | G | 0.405 | -0.0250 | 0.0036 | 6.65E-12 | 632802 | 47.1 |
| rs1160685 | 4 | 94052854 | G | C | 0.478 | 0.0208 | 0.0036 | 7.20E-09 | 632802 | 33.5 |
| rs6893752 | 5 | 60374912 | G | A | 0.766 | -0.0241 | 0.0041 | 3.25E-09 | 632802 | 35.0 |
| rs12186738 | 5 | 103816655 | T | G | 0.154 | -0.0333 | 0.0050 | 3.42E-11 | 632802 | 43.9 |
| rs1385108 | 5 | 154839646 | T | C | 0.239 | 0.0247 | 0.0042 | 3.00E-09 | 632802 | 35.2 |
| rs4044321 | 5 | 166989513 | G | A | 0.642 | -0.0278 | 0.0037 | 6.08E-14 | 632802 | 56.3 |
| rs4352629 | 5 | 87756821 | T | C | 0.492 | -0.0275 | 0.0036 | 1.22E-14 | 632802 | 59.5 |
| rs72789632 | 5 | 106834363 | T | C | 0.12 | -0.0329 | 0.0053 | 5.02E-10 | 632802 | 38.7 |
| rs9401770 | 6 | 98748008 | A | G | 0.273 | 0.0277 | 0.0040 | 3.47E-12 | 632802 | 48.4 |
| rs222449 | 6 | 52916062 | T | A | 0.793 | -0.0253 | 0.0044 | 1.08E-08 | 632802 | 32.7 |
| rs3800227 | 6 | 108994161 | G | A | 0.701 | 0.0228 | 0.0041 | 1.93E-08 | 632802 | 31.6 |
| rs10498846 | 6 | 67405337 | T | C | 0.473 | 0.0206 | 0.0036 | 6.62E-09 | 632802 | 33.6 |
| rs240963 | 6 | 111644332 | C | T | 0.836 | -0.0410 | 0.0048 | 2.16E-17 | 632802 | 72.0 |
| rs12333760 | 7 | 99185406 | C | T | 0.204 | -0.0290 | 0.0048 | 1.44E-09 | 632802 | 36.6 |
| rs10233018 | 7 | 117523709 | G | A | 0.503 | 0.0271 | 0.0036 | 2.75E-14 | 632802 | 57.9 |
| rs10279261 | 7 | 133589846 | A | G | 0.619 | -0.0214 | 0.0037 | 5.00E-09 | 632802 | 34.2 |
| rs10260968 | 7 | 1889773 | A | G | 0.597 | -0.0203 | 0.0036 | 1.75E-08 | 632802 | 31.7 |
| rs12112638 | 7 | 69735251 | G | A | 0.275 | -0.0245 | 0.0040 | 1.34E-09 | 632802 | 36.8 |
| rs4236259 | 7 | 1708080 | G | T | 0.499 | -0.0248 | 0.0036 | 3.35E-12 | 632802 | 48.5 |
| rs2140114 | 7 | 3407568 | T | C | 0.518 | -0.0233 | 0.0037 | 4.70E-10 | 632802 | 38.8 |
| rs3801289 | 7 | 96638267 | C | A | 0.351 | -0.0221 | 0.0037 | 3.74E-09 | 632802 | 34.8 |
| rs1565735 | 8 | 27426077 | A | T | 0.212 | -0.0376 | 0.0045 | 3.42E-17 | 632802 | 71.1 |
| rs1899896 | 8 | 93201036 | T | C | 0.286 | 0.0264 | 0.0039 | 1.04E-11 | 632802 | 46.3 |
| rs13261666 | 8 | 59814666 | T | G | 0.522 | -0.0269 | 0.0036 | 3.90E-14 | 632802 | 57.2 |
| rs12545053 | 8 | 65073605 | G | A | 0.397 | 0.0203 | 0.0036 | 2.43E-08 | 632802 | 31.1 |
| rs2631024 | 8 | 91995577 | G | A | 0.737 | -0.0230 | 0.0040 | 1.18E-08 | 632802 | 32.5 |
| rs4543592 | 9 | 3014254 | C | T | 0.468 | 0.0219 | 0.0036 | 7.46E-10 | 632802 | 37.9 |
| rs2378662 | 9 | 86707289 | A | G | 0.556 | 0.0209 | 0.0036 | 4.16E-09 | 632802 | 34.5 |
| rs10114490 | 9 | 11070165 | A | G | 0.198 | -0.0255 | 0.0045 | 1.81E-08 | 632802 | 31.7 |
| rs10905461 | 10 | 8803551 | C | T | 0.718 | -0.0240 | 0.0041 | 7.35E-09 | 632802 | 33.4 |
| rs7921378 | 10 | 63674885 | C | G | 0.463 | -0.0255 | 0.0036 | 8.26E-13 | 632802 | 51.2 |
| rs12356821 | 10 | 104563808 | C | G | 0.14 | 0.0394 | 0.0050 | 6.27E-15 | 632802 | 60.8 |
| rs10159545 | 10 | 21766969 | G | C | 0.375 | 0.0263 | 0.0037 | 1.84E-12 | 632802 | 49.6 |
| rs9423279 | 10 | 125680419 | G | C | 0.641 | -0.0205 | 0.0037 | 3.21E-08 | 632802 | 30.6 |
| rs7938812 | 11 | 112911004 | G | T | 0.424 | 0.0438 | 0.0036 | 2.71E-33 | 632802 | 145.0 |
| rs6265 | 11 | 27679916 | T | C | 0.203 | -0.0318 | 0.0046 | 3.77E-12 | 632802 | 48.2 |
| rs7929518 | 11 | 85980958 | G | A | 0.765 | 0.0242 | 0.0043 | 1.56E-08 | 632802 | 32.0 |
| rs4523689 | 11 | 7950797 | G | A | 0.408 | -0.0206 | 0.0036 | 1.55E-08 | 632802 | 32.0 |
| rs11057005 | 12 | 16748721 | G | A | 0.43 | -0.0209 | 0.0036 | 4.85E-09 | 632802 | 34.2 |
| rs4759228 | 12 | 56508409 | C | G | 0.27 | -0.0217 | 0.0039 | 3.58E-08 | 632802 | 30.4 |
| rs7969559 | 12 | 69655167 | G | A | 0.688 | -0.0244 | 0.0040 | 7.31E-10 | 632802 | 37.9 |
| rs1971318 | 12 | 121389500 | T | C | 0.141 | 0.0285 | 0.0049 | 7.06E-09 | 632802 | 33.5 |
| rs7322872 | 13 | 100548329 | T | C | 0.782 | -0.0256 | 0.0043 | 3.58E-09 | 632802 | 34.8 |
| rs3904512 | 13 | 38357471 | A | G | 0.429 | -0.0212 | 0.0036 | 3.23E-09 | 632802 | 35.0 |
| rs9540729 | 13 | 66947124 | T | A | 0.501 | -0.0196 | 0.0036 | 3.82E-08 | 632802 | 30.2 |
| rs76214862 | 14 | 29500130 | C | A | 0.202 | -0.0250 | 0.0045 | 3.99E-08 | 632802 | 30.2 |
| rs12441907 | 15 | 83922387 | A | C | 0.186 | -0.0292 | 0.0045 | 1.06E-10 | 632802 | 41.7 |
| rs1435741 | 15 | 47935843 | A | G | 0.425 | 0.0294 | 0.0036 | 2.64E-16 | 632802 | 67.1 |
| rs4785836 | 16 | 65604652 | C | T | 0.398 | -0.0205 | 0.0037 | 2.26E-08 | 632802 | 31.3 |
| rs7197072 | 16 | 717085 | T | C | 0.238 | -0.0248 | 0.0042 | 2.77E-09 | 632802 | 35.3 |
| rs1050847 | 16 | 87443734 | T | C | 0.505 | -0.0216 | 0.0036 | 1.67E-09 | 632802 | 36.3 |
| rs4781977 | 16 | 17572674 | C | T | 0.205 | -0.0239 | 0.0044 | 4.54E-08 | 632802 | 29.9 |
| rs11078713 | 17 | 7795972 | G | A | 0.454 | -0.0202 | 0.0036 | 2.23E-08 | 632802 | 31.3 |
| rs7224742 | 17 | 30657058 | T | C | 0.595 | -0.0207 | 0.0037 | 1.43E-08 | 632802 | 32.1 |
| rs11658881 | 17 | 2072949 | G | A | 0.418 | 0.0201 | 0.0036 | 2.43E-08 | 632802 | 31.1 |
| rs6508144 | 18 | 50026142 | G | C | 0.563 | -0.0207 | 0.0036 | 7.97E-09 | 632802 | 33.3 |
| rs11872397 | 18 | 72535282 | A | G | 0.252 | -0.0248 | 0.0041 | 1.43E-09 | 632802 | 36.6 |
| rs72896886 | 18 | 42632652 | C | G | 0.144 | -0.0269 | 0.0048 | 2.75E-08 | 632802 | 30.9 |
| rs76608582 | 19 | 4474725 | A | C | 0.0389 | -0.0496 | 0.0083 | 1.94E-09 | 632802 | 36.0 |
| rs1555445 | 20 | 31175258 | T | A | 0.337 | 0.0226 | 0.0038 | 3.65E-09 | 632802 | 34.8 |
| rs117143374 | 21 | 40555561 | C | T | 0.12 | 0.0293 | 0.0053 | 2.76E-08 | 632802 | 30.9 |
| rs134529 | 22 | 28781758 | C | T | 0.349 | -0.0200 | 0.0037 | 4.85E-08 | 632802 | 29.8 |

Supplementary Table 2. 23 SNPs associated with cigarettes per day.

| SNP | Chromosome | Position | Effect allele | Other allele | Frequency | Beta | Se | P | Sample size | F |
| --- | --- | --- | --- | --- | --- | --- | --- | --- | --- | --- |
| rs2072659 | 1 | 154548521 | G | C | 0.105 | -0.0653 | 0.0092 | 1.71E-12 | 225752 | 49.8 |
| rs2084533 | 3 | 16872929 | T | C | 0.319 | 0.0336 | 0.0059 | 1.22E-08 | 260706 | 32.5 |
| rs7431710 | 3 | 48935583 | A | G | 0.644 | -0.0350 | 0.0058 | 1.82E-09 | 261833 | 36.2 |
| rs787362 | 4 | 67904931 | A | T | 0.452 | 0.0305 | 0.0056 | 4.50E-08 | 262450 | 29.9 |
| rs11725618 | 4 | 67053769 | C | T | 0.287 | 0.0361 | 0.0062 | 4.67E-09 | 250822 | 34.3 |
| rs806798 | 6 | 26214473 | C | T | 0.543 | -0.0309 | 0.0055 | 2.48E-08 | 257120 | 31.1 |
| rs215600 | 7 | 32333642 | A | G | 0.64 | -0.0493 | 0.0058 | 1.10E-17 | 262746 | 73.3 |
| rs58379124 | 8 | 42579203 | C | T | 0.748 | 0.0669 | 0.0065 | 9.00E-25 | 258370 | 106.0 |
| rs790564 | 8 | 64604218 | C | A | 0.719 | -0.0409 | 0.0062 | 3.97E-11 | 261674 | 43.6 |
| rs73229090 | 8 | 27442127 | A | C | 0.113 | 0.0555 | 0.0088 | 2.44E-10 | 255628 | 40.1 |
| rs3025383 | 9 | 136502369 | C | T | 0.18 | -0.0578 | 0.0070 | 2.22E-16 | 256134 | 67.4 |
| rs75494138 | 11 | 46465361 | T | C | 0.0618 | 0.0599 | 0.0106 | 1.45E-08 | 258399 | 32.1 |
| rs7928017 | 11 | 113448762 | A | C | 0.413 | -0.0329 | 0.0056 | 3.14E-09 | 258763 | 35.1 |
| rs7951365 | 11 | 16377044 | C | T | 0.306 | 0.0390 | 0.0060 | 6.63E-11 | 258295 | 42.6 |
| rs632811 | 15 | 59155050 | G | A | 0.351 | -0.0367 | 0.0064 | 1.03E-08 | 214251 | 32.8 |
| rs8034191 | 15 | 78806023 | C | T | 0.328 | 0.1826 | 0.0059 | 1.00E-200 | 255729 | 961.0 |
| rs1579233 | 16 | 52074530 | G | A | 0.571 | -0.0318 | 0.0056 | 1.07E-08 | 259482 | 32.7 |
| rs4785587 | 16 | 89772619 | A | G | 0.511 | -0.0336 | 0.0055 | 1.27E-09 | 254249 | 36.9 |
| rs895330 | 19 | 4060707 | G | C | 0.206 | -0.0390 | 0.0070 | 2.68E-08 | 250059 | 30.9 |
| rs34406232 | 19 | 41305530 | A | C | 0.0259 | -0.1470 | 0.0167 | 1.33E-18 | 251880 | 77.5 |
| rs56113850 | 19 | 41353107 | C | T | 0.568 | 0.1072 | 0.0056 | 1.10E-81 | 243952 | 366.0 |
| rs2273500 | 20 | 61986949 | C | T | 0.159 | 0.0681 | 0.0078 | 2.47E-18 | 252983 | 76.3 |
| rs2424888 | 20 | 31047533 | A | G | 0.405 | 0.0335 | 0.0056 | 2.76E-09 | 257430 | 35.3 |

Supplementary Table 3. 101 SNPs associated with past tobacco smoking.

| SNP | Chromosome | Position | Effect allele | Other allele | Frequency | Beta | Se | P | Sample size | F |
| --- | --- | --- | --- | --- | --- | --- | --- | --- | --- | --- |
| rs2186122 | 1 | 66470206 | T | A | 0.559411 | -0.0156973 | 0.0027 | 9.00E-09 | 424960 | 33.1 |
| rs17503369 | 1 | 73847108 | C | T | 0.182208 | 0.0216357 | 0.0035 | 6.60E-10 | 424960 | 38.1 |
| rs77068442 | 1 | 158845316 | G | A | 0.107228 | -0.0239119 | 0.0044 | 4.10E-08 | 424960 | 30.1 |
| rs10914684 | 1 | 33795572 | A | G | 0.325484 | 0.0165925 | 0.0029 | 7.80E-09 | 424960 | 33.3 |
| rs2367724 | 1 | 44107428 | T | C | 0.673602 | 0.0160073 | 0.0029 | 2.40E-08 | 424960 | 31.1 |
| rs11165623 | 1 | 96893000 | A | G | 0.504148 | -0.0158064 | 0.0027 | 4.40E-09 | 424960 | 34.4 |
| rs35761479 | 1 | 154154194 | A | G | 0.120751 | 0.0229646 | 0.0041 | 2.80E-08 | 424960 | 30.8 |
| rs6588376 | 1 | 50602495 | A | G | 0.208312 | -0.0188335 | 0.0033 | 1.40E-08 | 424960 | 32.2 |
| rs3935790 | 1 | 208725458 | A | G | 0.416104 | -0.0154673 | 0.0027 | 1.60E-08 | 424960 | 31.9 |
| rs1040070 | 1 | 74977870 | C | G | 0.56882 | 0.0162775 | 0.0027 | 2.50E-09 | 424960 | 35.6 |
| rs147052174 | 1 | 179783167 | T | G | 0.018556 | -0.0603916 | 0.0100 | 1.40E-09 | 424960 | 36.7 |
| rs74676797 | 2 | 633063 | A | G | 0.80811 | -0.0233457 | 0.0035 | 3.60E-11 | 424960 | 43.8 |
| rs528301 | 2 | 45154908 | A | G | 0.554258 | -0.0203355 | 0.0027 | 5.60E-14 | 424960 | 56.5 |
| rs7582445 | 2 | 60495874 | C | A | 0.587784 | -0.0172231 | 0.0027 | 3.20E-10 | 424960 | 39.6 |
| rs7609050 | 2 | 156021862 | C | A | 0.525594 | -0.0157639 | 0.0027 | 5.00E-09 | 424960 | 34.2 |
| rs11693702 | 2 | 162802184 | A | T | 0.463056 | -0.0171646 | 0.0027 | 2.20E-10 | 424960 | 40.3 |
| rs67174662 | 2 | 59295476 | G | A | 0.375322 | 0.0166562 | 0.0028 | 2.30E-09 | 424960 | 35.7 |
| rs1492546 | 2 | 81013736 | G | C | 0.55292 | -0.0154554 | 0.0027 | 1.20E-08 | 424960 | 32.5 |
| rs7596680 | 2 | 146114071 | G | C | 0.535389 | 0.026939 | 0.0027 | 2.00E-23 | 424960 | 99.5 |
| rs7600005 | 2 | 48212055 | A | G | 0.373357 | 0.0156078 | 0.0028 | 2.60E-08 | 424960 | 31.0 |
| rs290882 | 2 | 118262987 | C | T | 0.245196 | 0.0174671 | 0.0031 | 2.30E-08 | 424960 | 31.2 |
| rs13009008 | 2 | 174043233 | G | A | 0.672659 | 0.0157552 | 0.0029 | 3.90E-08 | 424960 | 30.2 |
| rs6751705 | 2 | 104113702 | G | T | 0.512137 | -0.0224698 | 0.0027 | 7.10E-17 | 424960 | 69.6 |
| rs3811038 | 2 | 113240183 | C | T | 0.275964 | -0.0170544 | 0.0030 | 1.80E-08 | 424960 | 31.7 |
| rs67336646 | 3 | 85524474 | A | T | 0.626947 | 0.0236432 | 0.0028 | 1.80E-17 | 424960 | 72.3 |
| rs9835772 | 3 | 85766025 | T | A | 0.243587 | -0.0182732 | 0.0031 | 5.70E-09 | 424960 | 33.9 |
| rs12487411 | 3 | 34422170 | A | G | 0.470776 | 0.0167975 | 0.0027 | 5.00E-10 | 424960 | 38.7 |
| rs963354 | 3 | 157393770 | A | C | 0.67342 | -0.0176444 | 0.0029 | 8.70E-10 | 424960 | 37.6 |
| rs1499976 | 3 | 117801330 | C | T | 0.852678 | -0.0314256 | 0.0038 | 1.90E-16 | 424960 | 67.7 |
| rs56760958 | 3 | 85983138 | T | C | 0.390264 | 0.0172678 | 0.0028 | 3.80E-10 | 424960 | 39.2 |
| rs899631 | 4 | 57749363 | T | G | 0.391204 | 0.0176508 | 0.0028 | 1.80E-10 | 424960 | 40.7 |
| rs77304846 | 4 | 70495353 | C | T | 0.183007 | 0.0208584 | 0.0035 | 2.10E-09 | 424960 | 35.9 |
| rs58400863 | 4 | 31184484 | A | G | 0.341566 | 0.0176273 | 0.0029 | 6.50E-10 | 424960 | 38.2 |
| rs3827592 | 4 | 147948150 | A | G | 0.350953 | 0.0177215 | 0.0028 | 3.80E-10 | 424960 | 39.2 |
| rs6828849 | 4 | 173077123 | T | A | 0.418816 | 0.017917 | 0.0027 | 5.70E-11 | 424960 | 42.9 |
| rs1559278 | 5 | 50794221 | C | T | 0.361217 | 0.0154864 | 0.0028 | 3.40E-08 | 424960 | 30.5 |
| rs7733542 | 5 | 106410664 | G | A | 0.629381 | -0.0156617 | 0.0028 | 2.50E-08 | 424960 | 31.0 |
| rs1017998 | 5 | 79263211 | G | A | 0.620358 | -0.0151907 | 0.0028 | 4.40E-08 | 424960 | 30.0 |
| rs27003 | 5 | 94202167 | C | T | 0.695133 | -0.0164348 | 0.0029 | 2.20E-08 | 424960 | 31.3 |
| rs10474278 | 5 | 87841490 | G | A | 0.746763 | -0.0184912 | 0.0031 | 3.00E-09 | 424960 | 35.2 |
| rs4044321 | 5 | 166989513 | G | A | 0.641902 | 0.0183697 | 0.0028 | 6.90E-11 | 424960 | 42.6 |
| rs1611723 | 6 | 29830505 | G | A | 0.405117 | 0.0158409 | 0.0027 | 7.30E-09 | 424960 | 33.4 |
| rs4708899 | 6 | 157751114 | G | A | 0.575572 | 0.0158982 | 0.0027 | 6.20E-09 | 424960 | 33.8 |
| rs9381919 | 6 | 50931059 | T | G | 0.103581 | 0.0270628 | 0.0044 | 9.60E-10 | 424960 | 37.4 |
| rs2797793 | 6 | 37477262 | C | T | 0.603851 | 0.0152706 | 0.0028 | 3.00E-08 | 424960 | 30.7 |
| rs12209519 | 6 | 67549140 | G | A | 0.407575 | -0.0158856 | 0.0028 | 8.00E-09 | 424960 | 33.3 |
| rs9375371 | 6 | 98751680 | A | G | 0.269381 | -0.0198988 | 0.0030 | 6.30E-11 | 424960 | 42.7 |
| rs118202 | 6 | 111658371 | T | G | 0.817423 | 0.0324485 | 0.0035 | 1.20E-20 | 424960 | 86.8 |
| rs885011 | 7 | 3484778 | C | T | 0.501337 | 0.020272 | 0.0027 | 5.40E-14 | 424960 | 56.6 |
| rs1499300 | 7 | 132311000 | C | A | 0.157852 | 0.0217944 | 0.0037 | 3.60E-09 | 424960 | 34.8 |
| rs1899689 | 7 | 121964349 | T | C | 0.389116 | -0.0154222 | 0.0028 | 2.30E-08 | 424960 | 31.2 |
| rs12333760 | 7 | 99185406 | C | T | 0.165515 | 0.0213967 | 0.0036 | 3.90E-09 | 424960 | 34.7 |
| rs1174864 | 7 | 53127559 | A | G | 0.54986 | -0.0153603 | 0.0027 | 1.50E-08 | 424960 | 32.0 |
| rs6464024 | 7 | 1688369 | T | C | 0.427444 | 0.0175549 | 0.0027 | 1.10E-10 | 424960 | 41.6 |
| rs10233018 | 7 | 117523709 | G | A | 0.503043 | -0.0151439 | 0.0027 | 1.90E-08 | 424960 | 31.6 |
| rs73229090 | 8 | 27442127 | A | C | 0.117859 | 0.0288784 | 0.0042 | 8.40E-12 | 424960 | 46.7 |
| rs3857914 | 8 | 93184065 | C | T | 0.302135 | -0.0201533 | 0.0030 | 9.60E-12 | 424960 | 46.4 |
| rs2433055 | 8 | 133769805 | G | T | 0.447651 | 0.0148366 | 0.0027 | 4.60E-08 | 424960 | 29.9 |
| rs2952251 | 8 | 10143164 | G | A | 0.739866 | -0.0175299 | 0.0031 | 1.20E-08 | 424960 | 32.4 |
| rs10956808 | 8 | 92775372 | G | T | 0.422089 | 0.0189115 | 0.0027 | 4.90E-12 | 424960 | 47.7 |
| rs10959442 | 9 | 10993737 | G | T | 0.46641 | -0.0171998 | 0.0027 | 1.90E-10 | 424960 | 40.6 |
| rs1246265 | 9 | 86761745 | C | T | 0.695165 | -0.020922 | 0.0029 | 1.00E-12 | 424960 | 50.8 |
| rs28647734 | 9 | 137977033 | A | G | 0.210012 | -0.0191736 | 0.0033 | 8.30E-09 | 424960 | 33.2 |
| rs9299331 | 9 | 102146915 | C | T | 0.526134 | -0.0174941 | 0.0027 | 9.10E-11 | 424960 | 42.0 |
| rs9423279 | 10 | 125680419 | G | C | 0.657058 | 0.0164476 | 0.0029 | 1.30E-08 | 424960 | 32.3 |
| rs7901348 | 10 | 63679281 | G | T | 0.552343 | 0.0192949 | 0.0027 | 1.60E-12 | 424960 | 50.0 |
| rs12244388 | 10 | 104640052 | A | G | 0.338108 | -0.0255126 | 0.0028 | 3.30E-19 | 424960 | 80.2 |
| rs911773 | 10 | 123964980 | C | A | 0.489521 | 0.0152558 | 0.0027 | 1.50E-08 | 424960 | 32.1 |
| rs3808937 | 10 | 104230012 | T | C | 0.207824 | -0.020549 | 0.0033 | 6.20E-10 | 424960 | 38.2 |
| rs2862465 | 11 | 42379492 | A | G | 0.409159 | 0.0158416 | 0.0027 | 7.30E-09 | 424960 | 33.5 |
| rs2155290 | 11 | 112851068 | G | C | 0.38361 | -0.039464 | 0.0028 | 4.60E-46 | 424960 | 203.0 |
| rs540356 | 11 | 132203816 | A | C | 0.413629 | -0.0184455 | 0.0028 | 2.40E-11 | 424960 | 44.6 |
| rs6265 | 11 | 27679916 | T | C | 0.189039 | 0.0261462 | 0.0034 | 3.00E-14 | 424960 | 57.8 |
| rs71491831 | 11 | 124605783 | A | G | 0.075719 | 0.0305743 | 0.0051 | 2.00E-09 | 424960 | 35.9 |
| rs7969559 | 12 | 69655167 | G | A | 0.720515 | 0.0173419 | 0.0030 | 7.50E-09 | 424960 | 33.4 |
| rs597808 | 12 | 111973358 | G | A | 0.515766 | 0.0221177 | 0.0027 | 2.60E-16 | 424960 | 67.1 |
| rs11613961 | 12 | 133474880 | C | T | 0.086782 | -0.0293599 | 0.0048 | 9.30E-10 | 424960 | 37.5 |
| rs1109480 | 12 | 121083279 | A | G | 0.389103 | 0.0163393 | 0.0028 | 4.50E-09 | 424960 | 34.4 |
| rs56081685 | 13 | 59454140 | G | T | 0.31367 | 0.016504 | 0.0029 | 1.40E-08 | 424960 | 32.1 |
| rs837335 | 13 | 101180197 | C | G | 0.490322 | -0.0151088 | 0.0027 | 2.10E-08 | 424960 | 31.4 |
| rs7333559 | 13 | 100546450 | A | G | 0.788658 | 0.0199661 | 0.0033 | 1.80E-09 | 424960 | 36.2 |
| rs9542750 | 13 | 72393720 | C | T | 0.585734 | 0.0151549 | 0.0028 | 3.60E-08 | 424960 | 30.3 |
| rs12895462 | 14 | 77615441 | C | T | 0.191374 | 0.0200066 | 0.0034 | 6.30E-09 | 424960 | 33.7 |
| rs62022627 | 15 | 89904546 | G | A | 0.400649 | 0.0189626 | 0.0028 | 5.90E-12 | 424960 | 47.4 |
| rs8034783 | 15 | 47763726 | T | C | 0.101047 | -0.0282475 | 0.0045 | 3.30E-10 | 424960 | 39.5 |
| rs2289791 | 15 | 67476952 | T | G | 0.247319 | 0.0190853 | 0.0031 | 1.30E-09 | 424960 | 36.8 |
| rs763053 | 16 | 735921 | C | T | 0.226141 | 0.0194888 | 0.0032 | 1.70E-09 | 424960 | 36.3 |
| rs2866724 | 16 | 13760152 | G | A | 0.266293 | -0.0188049 | 0.0031 | 7.30E-10 | 424960 | 37.9 |
| rs77878475 | 16 | 18058548 | A | T | 0.084274 | 0.0330817 | 0.0050 | 4.80E-11 | 424960 | 43.3 |
| rs2917670 | 16 | 69758963 | C | T | 0.610217 | 0.0167548 | 0.0028 | 1.30E-09 | 424960 | 36.8 |
| rs35445224 | 16 | 73084276 | C | T | 0.181004 | -0.0206228 | 0.0036 | 8.10E-09 | 424960 | 33.3 |
| rs12450028 | 17 | 2207425 | T | C | 0.345053 | -0.0166053 | 0.0028 | 4.60E-09 | 424960 | 34.4 |
| rs7216173 | 17 | 51891405 | T | A | 0.782099 | 0.0207755 | 0.0033 | 3.40E-10 | 424960 | 39.4 |
| rs8071295 | 17 | 50131994 | A | C | 0.157348 | 0.0225071 | 0.0037 | 1.40E-09 | 424960 | 36.7 |
| rs2587507 | 17 | 77790135 | C | T | 0.505702 | 0.0155861 | 0.0027 | 5.90E-09 | 424960 | 33.9 |
| rs1623003 | 18 | 21165163 | T | C | 0.664037 | -0.0184743 | 0.0029 | 1.20E-10 | 424960 | 41.5 |
| rs12608052 | 18 | 49803160 | T | C | 0.518925 | 0.0161105 | 0.0027 | 2.40E-09 | 424960 | 35.6 |
| rs76608582 | 19 | 4474725 | A | C | 0.047264 | 0.0429722 | 0.0067 | 1.10E-10 | 424960 | 41.5 |
| rs6141314 | 20 | 31093514 | A | G | 0.241567 | -0.0191608 | 0.0032 | 1.50E-09 | 424960 | 36.6 |
| rs762995 | 22 | 42672124 | G | A | 0.535061 | 0.0148727 | 0.0027 | 3.70E-08 | 424960 | 30.3 |
| rs139896 | 22 | 38397797 | C | T | 0.64784 | -0.0162135 | 0.0028 | 9.00E-09 | 424960 | 33.1 |
